# Optimization of Stimulation Parameters for Targeted Activation of Multiple Neurons Using Closed-Loop Search Methods

**DOI:** 10.3390/pr5040081

**Published:** 2017-12-11

**Authors:** Michelle L. Kuykendal, Stephen P. DeWeerth, Martha A. Grover

**Affiliations:** 1 School of Electrical and Computer Engineering, Georgia Institute of Technology, Atlanta, GA 30332, USA; 2 Coulter Department of Biomedical Engineering, Georgia Institute of Technology and Emory University, Atlanta, GA 30332, USA; 3 Laboratory for Neuroengineering, Georgia Institute of Technology and Emory University, Atlanta, GA 30332, USA; 4 School of Chemical & Biomolecular Engineering, Georgia Institute of Technology, Atlanta, GA 30332, USA

**Keywords:** optimization, closed-loop, feedback, extracellular electrical stimulation, micro-electrode array (MEA), dissociated culture, Powell

## Abstract

Differential activation of neuronal populations can improve the efficacy of clinical devices such as sensory or cortical prostheses. Improving stimulus specificity will facilitate targeted neuronal activation to convey biologically realistic percepts. In order to deliver more complex stimuli to a neuronal population, stimulus optimization techniques must be developed that will enable a single electrode to activate subpopulations of neurons. However, determining the stimulus needed to evoke targeted neuronal activity is challenging. To find the most selective waveform for a particular population, we apply an optimization-based search routine, Powell’s conjugate direction method, to systematically search the stimulus waveform space. This routine utilizes a 1-D sigmoid activation model and a 2-D strength–duration curve to measure neuronal activation throughout the stimulus waveform space. We implement our search routine in both an experimental study and a simulation study to characterize potential stimulus-evoked populations and the associated selective stimulus waveform spaces. We found that for a population of five neurons, seven distinct sub-populations could be activated. The stimulus waveform space and evoked neuronal activation curves vary with each new combination of neuronal culture and electrode array, resulting in a unique selectivity space. The method presented here can be used to efficiently uncover the selectivity space, focusing experiments in regions with the desired activation pattern.

## 1. Introduction

By developing new techniques to selectively activate particular neurons within a population, stimulation devices can better control their direct effects on activated tissue, and thereby improve stimulus efficacy. Selective activation to modulate neuronal activity is crucial for many science and clinical applications because selectivity allows stimuli to target a specific population. In applications such as deep brain stimulation (DBS), which is used in treating Parkinson’s disease and epilepsy, targeted stimulation can guide a stimulus to alleviate symptoms due to disease or injury. A priority in designing stimuli is to reduce side effects resulting from the activation of off-target populations. During DBS, stimuli must be designed to specifically target a baseline activity level such that the stimulus evokes sufficient activity to provide a therapeutic effect, while not excessively activating tissue leading to side effects [1–4]. There is a therapeutic subspace in the strength–duration waveform space, between which side effects are reduced and stimulus efficacy is increased, and stimulation algorithms must incorporate feedback of the evoked activity to enable neuronal targeting within this subspace.

Improving the selectivity of electrical stimuli for targeted neuronal activation is also a critical step in the development of advanced neural prostheses. The prosthesis field is expansive, including peripheral and cortical prostheses, with applications including restoration of lost motor and sensory function in artificial limbs; cochlear prostheses for restoring audition [5,6]; retinal prostheses for restoring vision [7–9]; and cortical prostheses for inducing sensory percepts and reading motor intent directly from the brain [10–16]. An effective prosthesis must encode a variety of unique stimuli. For example, the hand senses surface texture, heat, pressure, and directionality of contact, all of which are encoded uniquely. An early pioneering team pursuing the development of selective stimulation techniques utilized the cable model to discover that monophasic cathodic stimuli could be used to selectively activate neuronal fibers over cell bodies [17]. There is a vast potential neuronal activation space available for exploitation to extend the repertoire of stimulus messages by activating various subpopulations of the accessible neuronal population. Studies have shown that by using cortical electrodes, patients are able to detect the activation of even a single neuron [18], suggesting even the smallest differences in the activated population of neurons are detectable.

Our goal is to develop a technique that facilitates the measurement of all accessible neuronal subpopulations and finds the waveforms most selective for a target group. Exploiting the spatial location and natural variation in stimulus-evoked activation probabilities assists in the preferential selection of neuronal populations. The activation probability, in response to a rectangular current-pulse, is described by a two-parameter strength–duration curve. Although a neuron will typically activate with greater probability as charge is increased, some neurons activate preferentially to a long pulse-width, while others respond preferentially to a short-pulse-width, high-amplitude pulse [19]. For any given pair of accessible neurons, the inherent differences in their strength–duration curves can be exploited for delivering selective waveforms. However, searching a multi-dimensional parameter space, which could include stimulus strength and duration, electrode location or multiple electrodes and number of neurons in a population is technically challenging.

Closed-loop (CL) methods are well suited for fast searches through a large input parameter space to find an optimal stimulus waveform, owing to their online feedback of measured responses for determining subsequent stimuli [20–23]. Closed-loop techniques are advantageous over open-loop techniques in a multi-parameter space because CL techniques can learn from past data to rapidly locate the stimulus space that provides the most selective neuronal activation. A model-based search routine can guide the search and mitigate the inherent noise in the stimulus-evoked neuronal response. By utilizing CL search methods, Brocker et al. [24] used a computational model and genetic algorithm to develop non-regular temporal stimulation patterns for DBS, which, when tested experimentally, improved stimulus efficacy while reducing device power requirements. Additionally, Pais-Vieira et al. [25] implemented a brain-to-brain interface in rats that altered the stimulus waveform in one cortical prosthesis based on the actions of a separate rat, and the pair of rats learned to change their behavior to benefit them both. Numerous other studies have been conducted to investigate selectivity of electrical stimuli in humans, including for example, an investigation of human nerve stimulation thresholds [26], selective stimulation for improvement in motion control of musculoskeletal systems [27], selective stimulation of the human femoral nerve [28] and the optimization of selective stimulation parameters for multi-contact electrodes [29]. These developments in science and technology, many of which were successful due to the adoption of closed-loop methodologies, are not limited to neuroscience. For example, McMullen and Jensen [30] developed a model-based multi-dimensional optimization of a microreactor that monitors a chemical reaction where no a priori information is available on the reaction parameters. By utilizing real-time feedback of an estimate of the system state, CL techniques can improve on current technologies by increasing search efficiency to find optimal input parameters.

In this work, we have implemented an automated search technique, Powell’s conjugate direction method, to traverse the input parameter space. The difference in strength–duration curves among neurons creates regions in the waveform space that offer access to stimulus selectivity. Adopting Powell’s method for optimizing stimulus parameters allows for multiple parameters to be probed simultaneously in order to find the maximum in selectivity. Deterministic optimization methods, such as Powell’s method, generally start with an initial guess, and then iteratively improve on the solution according to a directional search algorithm. Our application of Powell’s method allows us to rapidly search through multiple variables to maximize the difference between activation curves. Resistance to noise is a design priority, given that neuronal responses are inherently noisy, and Powell’s method is more resistant to noise than gradient approaches since taking the derivative of noisy data leads to inaccuracy.

## 2. Material and Methods

**Figure 1.**
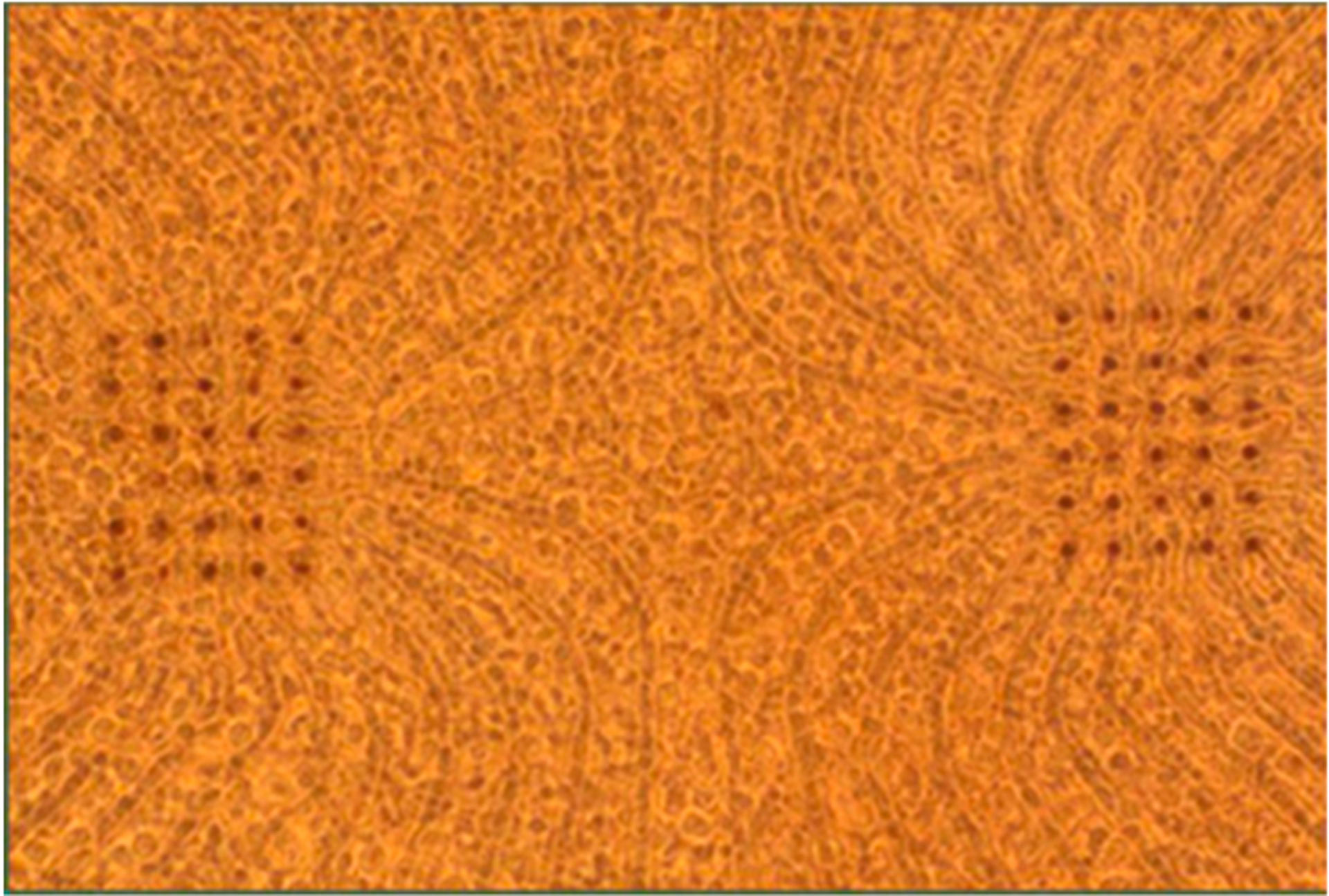
Phase contrast micrograph of the high-density electrode array, on which healthy neurons are growing. The HD array consists of two 6 × 5 electrode grids (10 μm diameter, 30 μm spacing). The distance from center-to-center of the two electrode arrays is 200 μm.

**Figure 2.**
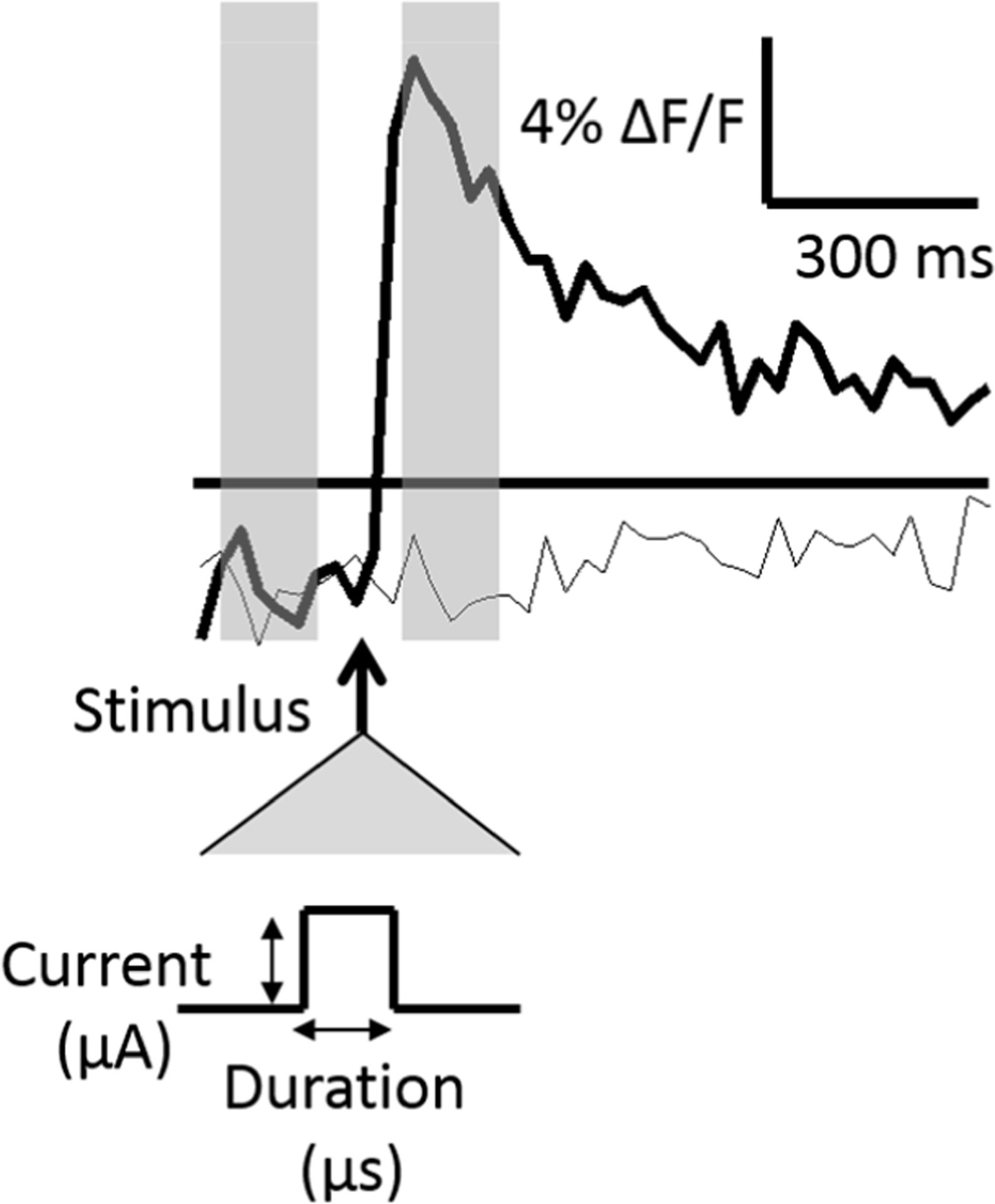
Stimulus-evoked fluorescence traces. Two traces are shown in which an action potential was evoked in response to the stimulus (bold line) and no action potential was evoked (light line). The stimulus timing with respect to the evoked signal is denoted by the bold arrow and is expanded below to show the two stimulus pulse control variables, the current (μA) and the pulse width, or duration (μs). Action potentials were assumed to occur if the post-stimulus change in fluorescence (ΔF/F) was greater than three times pre-stimulus levels (threshold shown as a horizontal bar). The pre-and-post-stimulus fluorescence levels were calculated as a time-average of four frames (represented with transparent gray bars).

**Figure 3.**
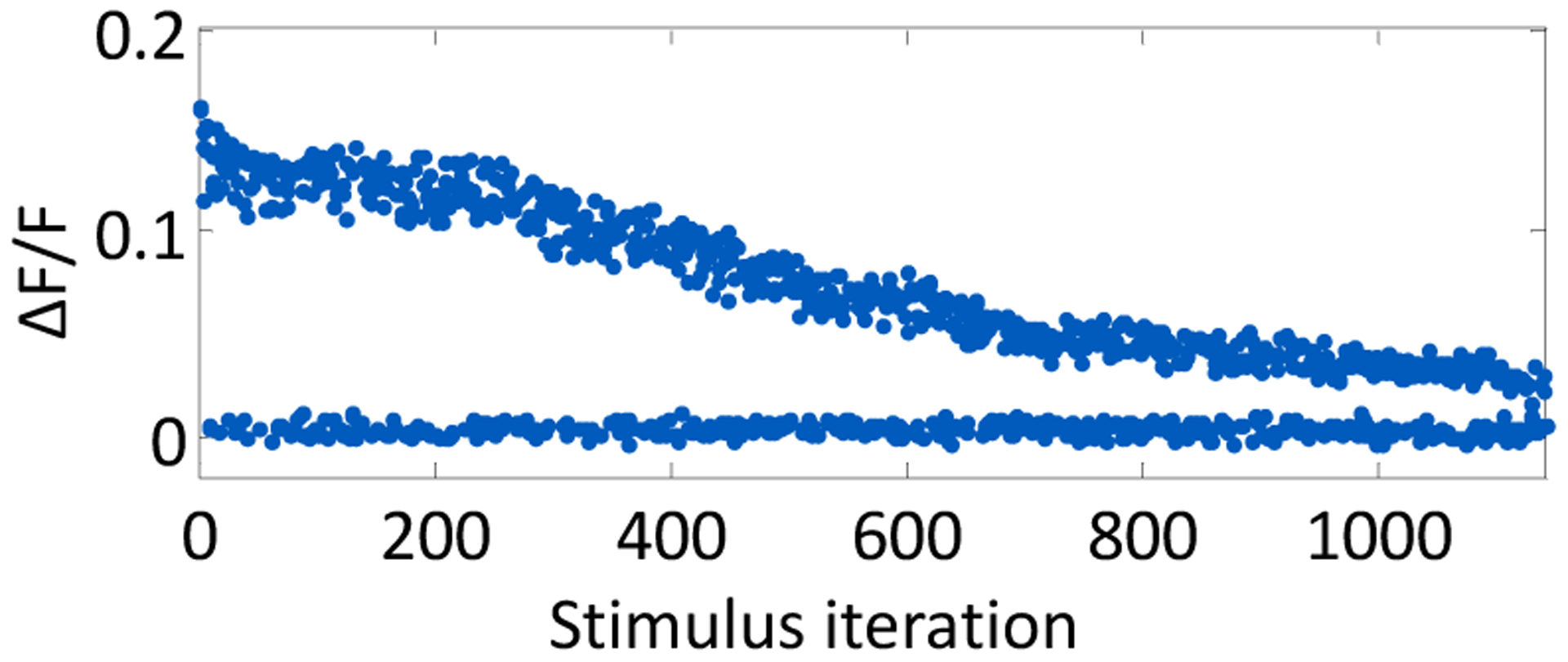
Evoked fluorescence decays due to photobleaching. The progression of the relative fluorescence change, ΔF/F, is shown across an experiment.

We designed a closed-loop system [31] for optimizing stimulus pulse parameters based on a model of neuronal activation and an experimental goal. Figures 1–3 have been reproduced from our previously published article [31] in order to allow the methods to be self-contained within this article. The system comprises hardware and software components that select and deliver stimuli designed to evoke a particular neuronal response. Each measured response is used to refine the model and the next stimulus is automatically chosen. The modular design, which separates data collection from both data analysis and decision-making, enables the user to select a model structure and an experimental goal in order to investigate a variety of questions. Each section of the system is described in more detail below.

### 2.1. Cortical Cell Culture

Embryonic Day 18 (E18) rat cortices were enzymatically and mechanically dissociated according to [32]. Cortices were digested with trypsin (0.25% w/EDTA) for 10–12 min, strained through a 40 μm cell strainer to remove clumps and centrifuged to remove cellular debris. Neurons were re-suspended in culture medium (90 mL Dulbecco’s Modified Eagle’s Medium (Irvine Scientific, Santa Ana, CA, USA, 9024), 10 mL horse serum (Life Technologies, Carlsbad, CA, USA, 16050-122), 250 μL GlutaMAX (200 mM; Life Technologies 35050-061), 1 mL sodium pyruvate (100 mM; Life Technologies 11360-070) and insulin (Sigma-Aldrich St. Louis, MO, USA, I5500; final concentration 2.5 μg/mL)) and diluted to 3000 cells/μL. Sixty electrode high density microelectrode arrays (MEAs; Multi Channel Systems, Reutlingen, Germany) were used for experimentation comprising 10 um TiN electrodes at a 30 um electrode spacing in a configuration of 2 grids of 6 × 5 electrodes. The MEA substrate was SiN and ITO (indium tin oxide) electrode tracks were chosen for their transparency during imaging. MEAs were sterilized by soaking in 70% ethanol for 15 min followed by UV exposure overnight. MEAs were treated with polyethylenimine to hydrophilize the surface, followed by three water washes and 30 min of drying. Laminin (10 μL; 0.02 mg/mL; Sigma-Aldrich L2020) was applied to the MEA for 20 min, half of the volume was removed, and 30,000 neurons were plated into the remaining laminin atop the MEA. A phase contrast micrograph of a culture atop a MEA can be seen in Figure 1. Cultures were protected using gas-permeable lids [32] and incubated at 35 °C in 5% carbon dioxide and 95% relative humidity. The culture medium was fully replaced on the first day in vitro (DIV) and then once every four DIV afterwards.

### 2.2. Electrical Stimulation

Electrical stimulation was performed using an STG-2004 stimulator and MEA-1060-Up-BC amplifier (Multi Channel Systems). MATLAB (Natick, MA, USA) was used to control all hardware devices, which were synchronized by TTL pulses sent from the stimulator at the beginning of each stimulation loop. In all stimulus iterations, a trigger pulse was first delivered to the camera to begin recording so that background fluorescence levels could be measured. An enable pulse was then delivered to the amplifier, which connected the stimulus channel to a pre-programmed electrode. A single cathodic square current pulse was then delivered to a single electrode centered under the camera field of view. Cathodic pulses were chosen because they have been shown to be most effective at evoking a neuronal response [33].

### 2.3. Optical Imaging

As described by Kuykendal et al. [31] automated optical imaging was used to measure the stimulus-evoked neuronal response. All preparation procedures were conducted in the dark to lengthen experiments by minimizing photobleaching and phototoxicity. First, culture media was removed and neurons were loaded with Fluo-5F AM (Life Technologies F-14222), a calcium-sensitive fluorescent dye with relatively low binding affinity at a concentration of 9.1 μM in in DMSO (Sigma-Aldrich D2650), Pluronic F-127 (Life Technologies P3000MP) and artificial cerebral spinal fluid (aCSF; 126 mM NaCl, 3 mM KCl, 1 mM NaH_2_PO_4_, 1.5 mM MgSO_4_, 2 mM CaCl_2_, 25 mM D-glucose) with 15 mM HEPES buffer for 30 min at ambient 25 °C and atmospheric carbon dioxide. Before imaging, cultures were rinsed two times with aCSF to remove free dye. Cultures were bathed in a mixture of synaptic blockers in aCSF (15 mM HEPES buffer). This included (2*R*)-amino-5-phosphonopentanoate (AP5; 50 μM; Sigma-Aldrich A5282), a NMDA receptor antagonist; bicuculline methiodide (BMI; 20 μM; Sigma-Aldrich 14343), a GABAA receptor antagonist; and 6-cyano-7-nitroquinoxaline-2,3-dione (CNQX; 20 μM; Sigma-Aldrich C239), an AMPA receptor antagonist. This cocktail was shown to suppress neuronal communication [34] to ensure that the recorded neuronal activity was directly evoked by the stimulus. The culture was then kept in a heated amplifier (Multi Channel Systems TC02, 37C) within the imaging chamber. The stage position was calibrated with respect to the desired field of view using the electrodes as fiducial markers. A MATLAB graphical user interface was used to automatically position the field-of-view over the stimulation electrode. During an experiment neurons were illuminated using a light-emitting diode (LED; 500 nm peak power) and LED current source (TLCC-01-Triple LED; Prizmatix, Givat-Shmuel, Israel) through a 20× immersion objective and a fluorescein isothiocyanate (FITC) filter cube. Evoked activity was optically recorded using a high-speed electron multiplication CCD camera (30 fps; QuantEM 512S; Photometrics, Tucson, AZ, USA), which has a 512 × 512 pixel grid covering a 400 μm × 400 μm area. After an experiment concluded, three aCSF washouts were performed at three-minute intervals, the culture media was replaced, and the culture was returned to the incubator.

### 2.4. Detecting Action Potentials

For each neuron, the measured intensity of a 16 × 16-pixel (12.5 μm × 12.5 μm) field centered on the soma was spatially averaged. Calcium signaling is dynamic and continuous within both neurons and glia associated with a neuronal population; therefore, there exists a low-level fluorescence that can be measured within these cell bodies due to the action of the calcium indicator as a chelator trapped with all cells. However, numerous studies have been published demonstrating the use of calcium indicators to infer neuronal spiking enabled by both the relatively fast and large change in measurable fluorescence at a neuronal cell body immediately following an action potential [35–38].

The relative change in fluorescence, ΔF/F, was calculated by subtracting the baseline (an average of four pre-stimulus frames, 30 fps) from an average of four post-stimulus frames (30 fps) and dividing the difference by the baseline. The post-stimulus frames were defined as those immediately following the delivered stimulus. Two fluorescence traces are shown across time in Figure 2.

Figure 2 shows two traces, one in which an action potential was generated, and one in which no action potential was generated. The standard deviation of the baseline frames was calculated in initial stimulus iterations and used as a measure of the fluorescence noise level. An action potential was assumed to have occurred if the ΔF/F was greater than three times the noise level within a particular neuron.

The average decay time constant of a stimulus-evoked fluorescence curve was 1.5 s. Because of this relatively slow signal decay, the experiment loop time was chosen to be 4.5 s (three decay time constants) to allow the signal sufficient time to return to baseline. The progression of ΔF/F for one neuron over the course of 1140 open-loop stimulus iterations is plotted in Figure 3, which illustrates the evoked signal decay with increasing light exposure. Stimuli were randomly presented from a range of stimulus strengths and durations, such that the neuronal response is mixed throughout the experiment. For the first 200 stimuli, the change in fluorescence resulting from an evoked action potential is unchanging. The signal then subsequently decays with each light exposure.

### 2.5. The Sigmoidal Activation Model

A saturating nonlinear curve was used to fit to the neuronal probability of firing an action potential in response to a varying stimulus current or pulse width. Specifically, a two-parameter sigmoid was used to describe this 1-D activation curve for cathodic square-pulse stimuli:

(1) $$p=\frac{1}{1+b_{2}e^{-\left(x-b_{1}\right)}}$$

The sigmoid model provides an approximation for the stimulus needed to activate a particular neuron with a given probability. The input activation parameter, *x*, is either the stimulus current or pulse width, and the output is the probability, *p*, for a neuron to fire an action potential. The two parameters describing the sigmoid are *b*_1_, the midpoint of the sigmoid, and *b*_2_, the slope of the curve at the midpoint. Because the sigmoid describes a probability of activation, it spans from zero to one. Each stimulus delivered produces a binary output, either the neuron fired an action potential or it did not, however, along the transition region of the sigmoid curve, the same stimulus level that is delivered 10 times may have a 50% probability of activating the neuron under test. Previous work showed that the sigmoid curve is good fit for modeling neuronal activation [31].

### 2.6. Algorithm for Building One Dimensional Sigmoidal Activation Curves

The closed-loop search procedure was divided into two halves: First, the collected stimulus-response data set was fit to the sigmoid model, and second, the sigmoid model was used to calculate the next stimulus to be delivered. The algorithm always first began with five open-loop stimuli that divided the stimulation space evenly before any curve fitting was performed. After the fifth iteration, the sigmoid model was analytically linearized, and a linear least-squares fit of the midpoint and slope parameters was performed to calculate a reasonable guess for the two sigmoid parameters—the sigmoid midpoint and the slope of the curve at the midpoint. All measured stimulus-evoked responses were equally weighted as zeros and ones. The output of the linear regression was used as an initial guess for a nonlinear least squares curve fit using the MATLAB Optimization Toolbox, which generated the best-fit sigmoid parameters. At this point, the sigmoid model has been fit to the dataset. The next stimulus value was then chosen in order to gain information about the sigmoid model midpoint and slope. To do this, the algorithm was designed to deliver the next stimulus along the slope of the sigmoid curve. A target neuronal activation probability goal was randomly chosen from the set of 0.25, 0.50 and 0.75, which spans the linear transition region of the sigmoid. The stimulus that was predicted to produce the firing probability goal was calculated using the sigmoid fit parameters and the activation probability goal. When a neuronal activation sigmoid had a nearly infinite slope, which was often the case when the dataset was still small early on in the experiment, the next stimulus chosen would be the same as the previously delivered stimulus. To ensure that the algorithm did not get stuck at one stimulus value, a random jitter was added to the stimulus up to 20% in either direction so that more data would be collected over the full range of the transition region of the activation curve. Stimulus currents and pulse widths were binned into 0.2 μA and 20 μs blocks, respectively. After every stimulus iteration, the linear and nonlinear curve-fits were run to update the model. All stimulus-evoked responses that were collected were included in the model, and data were never discarded. The search algorithm is presented below in pseudo-code form.

1. Collect data for five distinct stimulus levels.
2. Fit the sigmoid model to all available data points (zeros and ones).
  - Fit the linearly transformed sigmoid model to all zeros and ones in the dataset.
    - Use the linearly transformed sigmoid model, which derives from Equation (1), to solve for the fit parameters *b*_1_ and *b*_2_.
      $$x=-\text{ln}\left[(1/p-1)/b_{2}\right]+b_{1}$$
  - Use the linear fit parameters as an initial guess for a nonlinear curve fit of the model in Equation (1)
    - Minimize the sum-squared error
    - Use lsqcurvefit Matlab algorithm to calculate *b*_1_ and *b*_2_
3. Select the stimulus parameter for the next step
  - Select from the set of probabilities {0.25, 0.50, 0.75} using randperm Matlab function
  - Calculate from the sigmoid model the corresponding stimulus value
    - Solve the linearly transformed sigmoid model described above, which derives from Equation (1), for *x*—the next stimulus value.
  - If stimulus value is same as previous step, add jitter up to 20% in stimulus value, according to a uniform random distribution.
4. Apply the calculated stimulus value in the experiment
  - Use calcium imaging and image processing determine if the stimulus-evoked change in measured fluorescence surpassed threshold.
5. If convergence is not reached or if stimulus step count is not met, return to Step 2, else stop

### 2.7. The Strength–Duration Activation Model

Probabilistic neuronal activation in the 2-D strength–duration waveform space was described according to Lapicque [39]:

(2) $$I=r\left(1+\frac{c}{PW}\right)$$

The stimulus pulse width, *PW*, is the input; the stimulus current, *I*, is the output, and the two model parameters are the rheobase, *r*, and the chronaxie, *c*. The rheobase describes the stimulus current below which a stimulus with infinite pulse width will not evoke an action potential, and the chronaxie describes the stimulus pulse width that corresponds to a stimulus current of twice the rheobase. A strength–duration curve must be defined for a particular activation probability, such that there are a set of non-intersecting probability strength–duration curves spanning the two-parameter waveform space.

### 2.8. Slices Through the Strength–Duration Waveform Space

**Figure 4.**
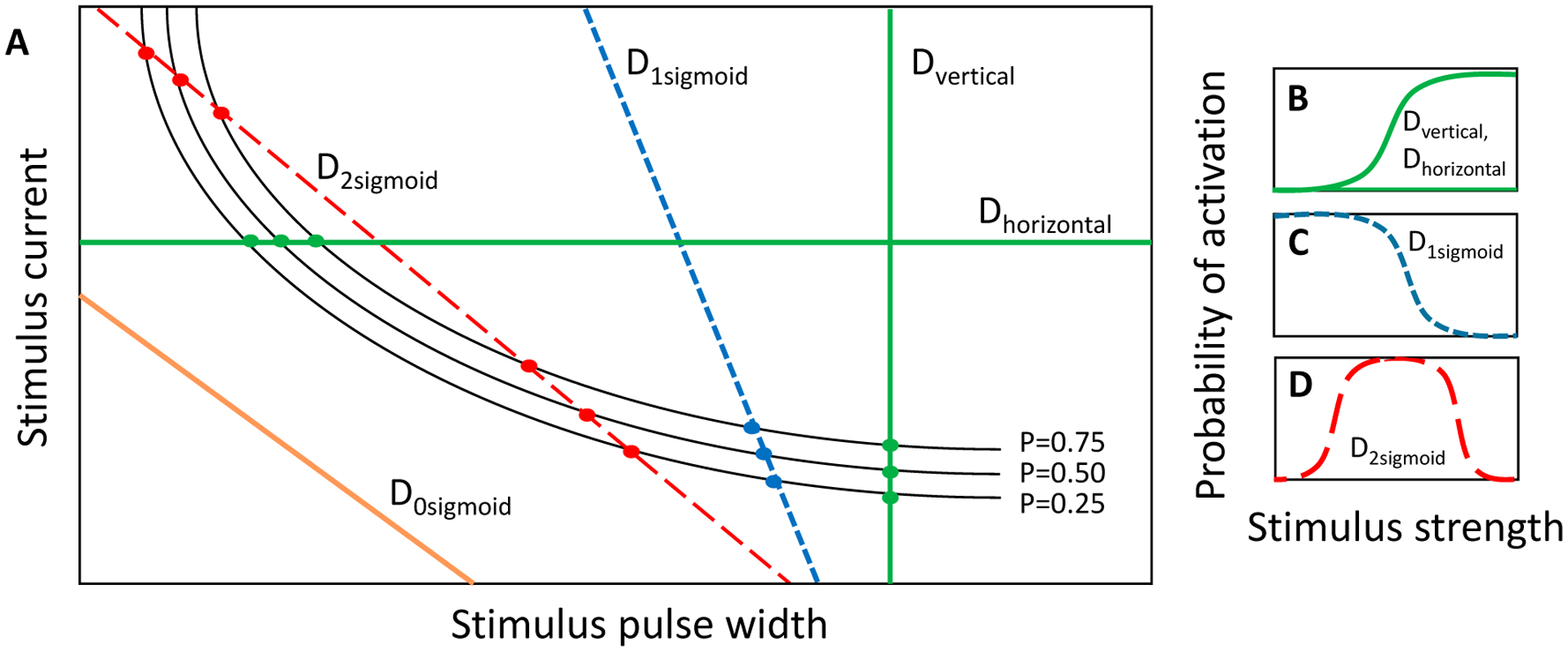
(**A**) Cartoon depiction of vertical (D_vertical_), horizontal (D_horizontal_), and negatively-sloped cross sections (D_0sigmoid_, D_1sigmoid_, D_2sigmoid_) through the strength–duration waveform space; (**B**) The vertical and horizontal slices cross the set of SD curves once, which is modeled by a positively sloped sigmoid. (**C**,**D**) The negatively-sloped slices can cross the set of SD curves either zero times (model not shown), which produces a zero probability of firing across the range of stimulus inputs; one time (**C**); which is modeled by a single negatively-sloped sigmoid; or two times (**D**); which is modeled by a probability space comprising a lower-threshold positively-sloped sigmoid and a higher-threshold negatively-sloped sigmoid.

We previously showed that when a one-dimensional slice is taken through the SD waveform space in either the horizontal or vertical direction, the activation probability could be modeled by a sigmoidal activation curve according to Equation (1) [31]. These slices are equivalent to constant-current and constant-pulse width curves, respectively, and are demonstrated by the green lines in Figure 4A,B. In a similar regard, slices can be taken along a positively sloped diagonal, and in that case, the stimulus strength is a combination of the stimulus current and pulse-width, but the sigmoid describing activation probability can be plotted as a function of either parameter, given that the slice line is defined to relate the two parameters. However, a slice with negative slope in the SD waveform space comprises a set of either zero, one or two sigmoidal activation curves depending on the number of times that the slice intersects with the probabilistic SD curves (Figure 4A). If all values of the input parameters lying along the slice fall below the probabilistic SD curves, then the activation model is zero for the entire slice. If the slice intersects once with the set of probability SD curves, then the activation model comprises a single sigmoid (Figure 4C). The sigmoidal parameters are estimated by fitting Equation (1) to the points where the slice intersects with the probabilistic SD curves. Lastly, if the slice intersects with the SD curves twice, once along the left-hand portion of the SD curves and once along the right-hand portion, then the activation probability first increases through the first crossing and then decreases through the second crossing. The activation model for the negatively sloped slice comprises the addition of two sigmoids: a lower-threshold sigmoid with positive slope and a higher-threshold sigmoid with negative slope (Figure 4D). It is important to note that for monophasic cathodic current-controlled stimuli, neurons will activate according to the strength–duration curve, and the slicing through the waveform space is only a means by which searches can be performed. In the case of the negatively sloped D2sigmoid (red dotted line in Figure 4A,D) neuronal activation is still considered on the probability scale of zero to one. In the search routine implemented in these studies, three discrete probability SD curves, having probabilities of 0.25, 0.50 and 0.75, were used for constructing the activation models along a slice through the SD waveform space (Figure 4A).

### 2.9. Powell’s Conjugate Direction Method Search Routine

**Figure 5.**
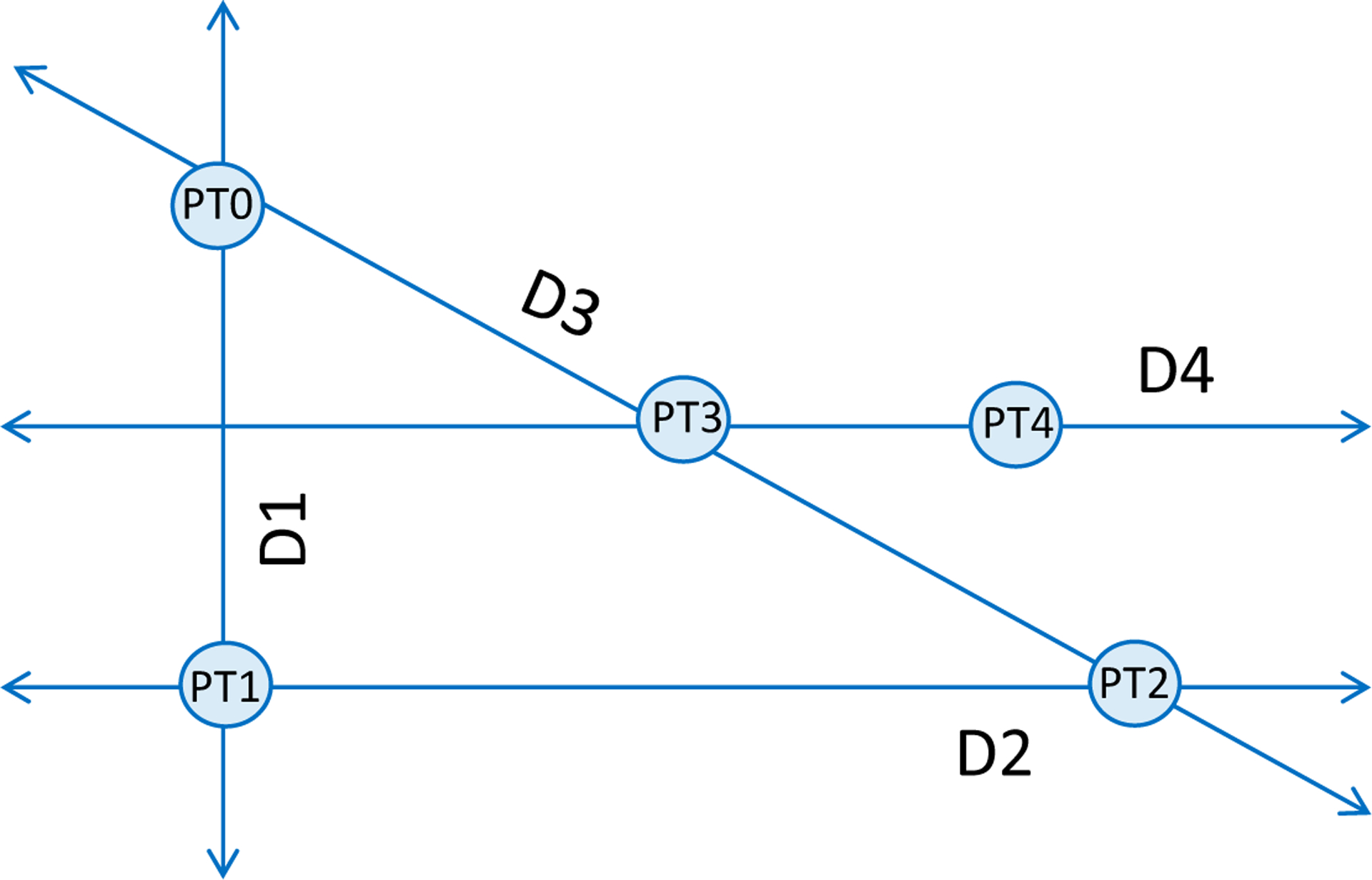
Depiction of the first four searches in Powell’s method. An initial point (PT0) is chosen in the 2-D search space. A search is performed in the vertical direction, D1, locating the maximal selectivity at PT1. Point PT1 becomes the starting point for a search orthogonal to the first search in direction D2. The maximal selectivity of the second search is found at PT2. The third search is performed in the direction connecting points PT0 and PT2, direction D3, and resulting in a new maximum PT3. The search continues with another horizontal search parallel to D2 and intersecting PT3; a subsequent search is performed in the direction that connects the newly found point, PT4, to PT2. The algorithm iterates until the search goal reached.

Powell’s conjugate direction method is a non-gradient search routine for finding the maximum (or minimum) of a function. It is especially applicable to multi-dimensional searches of noisy systems since its calculations do not rely upon derivatives, which are sensitive to noise. Powell’s method specifically dictates the direction of each search iteration through the input parameter space, which in this study is the strength–duration stimulus space, comprising a stimulus current and pulse-width for a rectangular pulse. An illustration of the generic search routine is depicted in Figure 5, which consists of a series of line searches through the input space. Each line search comprises one execution of the methods described above, in which the sigmoidal activation models are constructed for each of the neurons within the population. Along each line search, an objective function is evaluated. For this study, the objective function, *f*, measures the differences in sigmoidal activation curves such that the sum of off-target neuronal activation probabilities, for *m* neurons, is subtracted from the sum of target neuronal activation probabilities, for *n* neurons.

(3) $$f=\sum_{i=1}^{n}P_{i}-\overset{m}{\underset{j=1}{\sum }}P_{j}$$

The sigmoidal activation curves for the target population are summed such that as each target neuron activates, and the probability of firing transitions from zero to one, the objective function increases by one. As the off-target neurons begin to activate, and their probabilities of firing transition from zero to one, the objective function decreases by one. Therefore, along each line search, once all sigmoidal activation curves have been estimated, the on- and off-target activation curves are combined, and the maximum of the objective function is found.

Powell’s method begins with an arbitrary point, PT0, chosen from the input space. The first search direction (D1) is a vertical search crossing through PT0, which spans the extent of the space. For this implementation, D1 is a variable-current, fixed-pulse-width search bracketed by a minimum current of 0 μA and a maximum current of 25 μA. The maximum of the function is found at PT1, which is a measurement of the selectivity achievable between two neurons. Point PT1 corresponds to the peak of the difference between the two neuronal activation curves, both modeled as sigmoids. Like PT1, all following points found during a search also correspond to the maximum of the objective function, which we have defined as the absolute value of the difference of sigmoid activation curves. The next search direction, D2, is perpendicular to D1 and crosses through point PT1. The search for the maximum of the selectivity curve (PT2) is repeated, but in this case, the search is a variable-pulse-width, constant-current search, which spans the entire pulse-width space. After the first two searches, the routine alternates between diagonal and horizontal searches. Search direction 3, D3, is a multi-dimensional search in both current and pulse-width that passes through points PT0 and PT2. When the maximum of this search is found at PT3, the next search commences in direction D4, which passes through PT3 and is parallel to D2. The following search is in the direction that connects points PT2 and PT4. The search routine continues until the search goal is met. The implemented Powell method is presented below in pseudo-code form.

1. Conduct line search, *n*, to find stimulus parameters that maximize difference in neuronal activation probabilities
  - Determine the waveform space search direction
    - Case *n* = 1: Search is vertical through the waveform space with constant stimulus duration (pulse-width)
      1. Stimulus duration is pre-determined from setting “PT0”
    - Case *n* = 2, 4, 6 … : Search is orthogonal to the first case through the waveform space with constant stimulus current (amplitude)
      1. Stimulus current is determined from “PT(*n* − 1)”
    - Case *n* = 3, 5, 7 … : Search is diagonal through the waveform space, both stimulus current and duration are varied
      1. Direction is determined from line fit to “PT(*n* − 1)” and “PT(*n* − 3)”
  - Build activation curve models for neurons 1 to *n*
  - Evaluate the objective function: Find the maximum of the difference between neuronal activation curves
    - Define the next search result as a point within the stimulus waveform space with coordinates (stimulus duration, stimulus current), labeled “PTn”
2. If convergence is not reached based on the evaluation of the objective function, or a search count is not met, return to Step 1, else stop

### 2.10. Simulations of Powell’s Conjugate Direction Method Search Routine

To augment the experimental studies, simulated neuronal activation models were generated, using experimentally identified strength–duration curves for each neuron in the population. To implement Powell’s method, a starting point was chosen from the input parameter space, similarly to what was described in the experimental study above. A “true” sigmoidal activation model was then constructed for each neuron along the first line search, in either the vertical or horizontal direction. This “true” sigmoidal activation curve was then estimated in simulation similarly to the experimental study. The closed-loop routine for building sigmoidal activation models was executed, delivering 50 simulated stimuli through the one dimensional input parameter space, and a simulated model of the activation sigmoid was defined for each neuron in the study. These simulation studies enabled consideration of additional scenarios and objective functions for the same population of neurons. In reality, the number of experiments that can be performed is limited, due to photobleaching.

## 3. Results

To selectively activate a subpopulation of neurons, we experimentally implemented Powell’s method, using a series of model-based line searches to locate the optimal combination of stimulus strength and duration. In the first study, we applied our system to an experimental setting of cultured neurons to analyze the selectivity achievable between two neurons. We then extended this study by experimentally measuring strength–duration curves for a population of five cultured neurons. The selectivity space was mapped for the five neurons and the CL search routine was then used in simulation to conduct further studies based on models generated by the experimentally derived neuron activation curves. In the simulation studies, the robustness of the CL search method was explored to target subpopulations consisting of multiple neurons.

### 3.1. Powell’s Method Applied Experimentally to Find the Most Selective Waveform between a Pair of Neurons

Five iterations of Powell’s method were experimentally performed to find the most selective waveform between two neurons, labeled N1 and N2. In all five line searches, activation curves were constructed using the sigmoid model in Equation (1). Stimulus-evoked responses were collected at each stimulus point, and the sigmoid model was fit to all available data for each neuron according to the methods. The search algorithm applied 50 stimuli designed for each neuron, along the line defined online by the closed-loop search routine. As the stimulus space was divided and binned into 0.2 μA and 20 μs resolution blocks according to the methods for building one-dimensional sigmoid activation curves, numerous stimuli were delivered at each stimulus value. In this experiment, the 50 stimuli were delivered in order to construct a one-dimension activation curve, and after each search was performed for both neurons, the difference between the activation curves for N1 and N2 was determined according to Equation (3) and a maximal selectivity point was calculated in real time.

**Figure 6.**
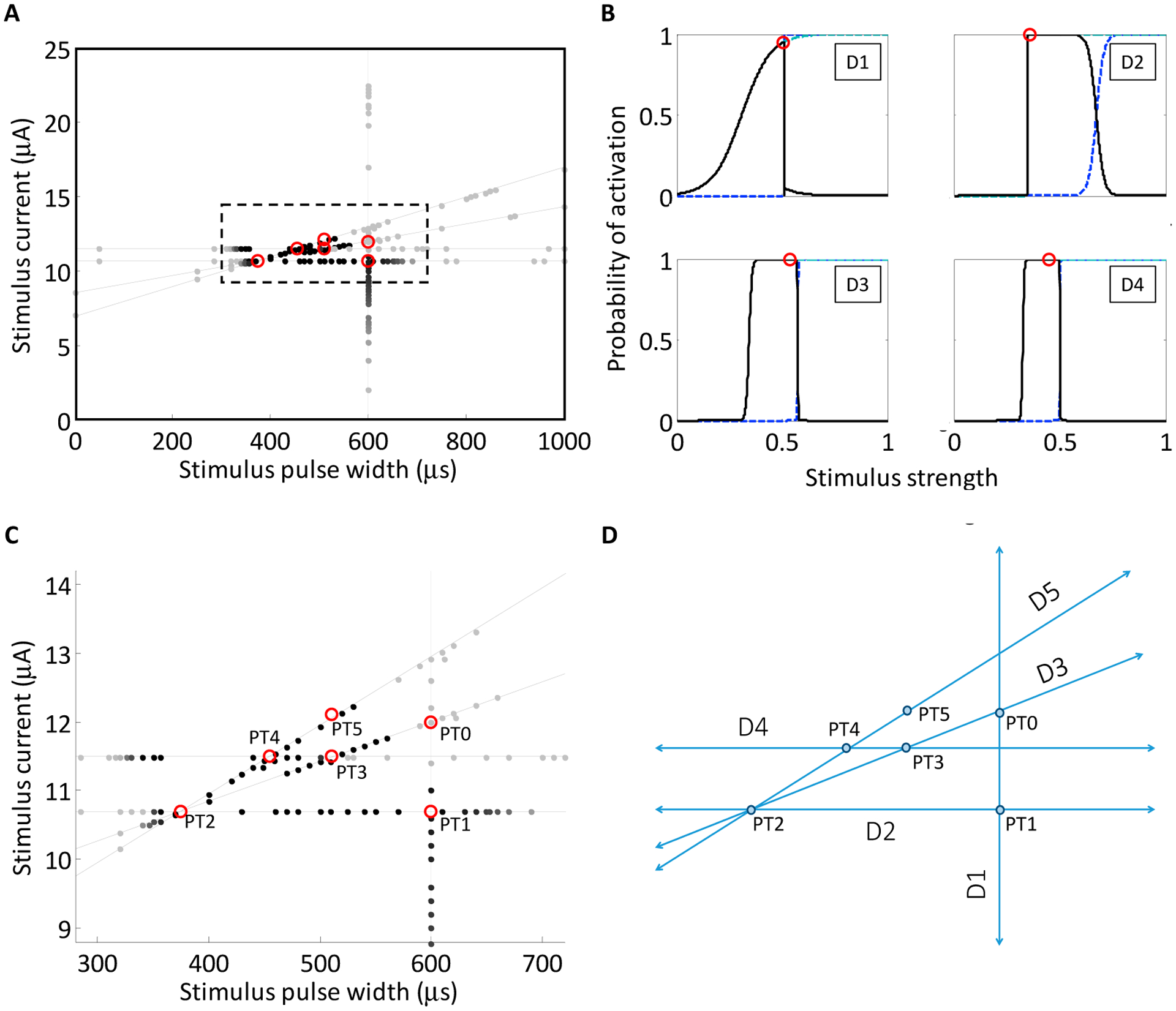
(**A**) Implementation of Powell’s method to search the strength–duration waveform space. The thin lines denote the five search segments. The stimuli applied along each stimulus path is color coded such that the darker the point, the greater the selectivity between the two neurons. The maxima found along the search lines are highlighted with open circles; (**B**) the objective function (solid line) was evaluated along each line search in the search routine of (**A**) according to Equation (3). The objective function for each of the first four searches is plotted in each panel, and the maximum of the objective function is denoted with an open circle, similarly to (**A**). The sigmoidal activation functions for each of the two neurons, N1 and N2, are plotted with dotted lines. The outputs from the first four are depicted here. In all five searches, activation curves for neurons N1 and N2, dotted lines, were estimated from data; (**C**) The implementation of the search routine, magnified from the dashed box in panel (**A**). The starting point, PT0, was chosen near the middle of the range of stimulus currents and pulse widths (600 μs, 12.0 μA); (**D**) a cartoon depiction of the search routine, shown in panel (**C**). Each of the search directions and measured peak selectivity points is highlighted.

The search routine automatically executed five complete multi-neuron search routines beginning from a starting point near the middle of the range of stimulus currents and pulse widths (600 μs, 12.0 μA), denoted as PT0 in Figure 6A. The first search was a stimulus current search from the starting point (D1, Figure 6A), with the stimulus pulse width fixed at 600 μs. This appears as a vertical line in Figure 6. The maximum of the objective function *f*, which is the difference in sigmoids produced by neurons N1 and N2, was calculated online to have occurred at 10.7 μA, and is depicted as point PT1. The sigmoid parameters (*b*_1_, *b*_2_) derived from the first search were (11.2 μA, 20.1 μA^−1^) for N1 and (6.80 μA, 0.67 μA^−1^) for N2. According to the Powell search method, the second search was a perpendicular (Figure 6C), horizontal stimulus pulse-width search (D2) crossing through PT1. The current was fixed at 10.7 μA, and the sigmoid search spanned the range of durations from 0 to 1000 μs. Again, the maximum of the difference of sigmoids for neurons N1 and N2 was calculated online to be located at 375 μs, point PT2 (375 μs, 10.7 μA). The sigmoid parameters (*b*_1_, *b*_2_) derived from the second search were (673 μs, 0.06 μs^−1^) for N1 and (349 μs, 3.06 μs^−1^) for N2. The third search direction was then calculated by the routine as a line connecting points PT0 and PT2 (Equation (4)), where *I* is the current (μA) and *PW* is the pulse width (μs).

(4) $$I=5.77\times 10^{-3}PW+8.53$$

The algorithm continued the automated search process. The activation curves were measured along the third search direction and the difference in sigmoids was again calculated. The maximum was measured at a pulse-width of 511 μs and current of 11.5 μA. The sigmoid parameters (*b*_1_, *b*_2_) derived from the third search were (574 μs, 0.75 μs^−1^) for N1 and (343 μs, 0.15 μs^−1^) for N2. The fourth search direction was then conducted parallel to the horizontal pulse-width search. The current was fixed from the previous point at 11.5 μA, and the stimulus pulse-width was allowed to vary through the entire range from 0 to 1000 μs. The maximum of the difference of sigmoids for neurons N1 and N2 was measured as point PT4, at 455 μs and 11.5 μA. The sigmoid parameters (*b*_1_, *b*_2_) derived from the fourth search were (501 μs, 1.05 μs^−1^) for N1 and (326 μs, 0.30 μs^−1^) for N2. The fifth and final selectivity search was a two-parameter diagonal search connecting points 2 and 4 along the line defined in Equation (5). The maximum difference between sigmoids was measured at a pulse width of 524 μs and a current of 12.1 μA. The sigmoid parameters (*b*_1_, *b*_2_) derived from the fifth search were (550 μs, 0.56 μs^−1^) for N1 and (345 μs, 0.41 μs^−1^) for N2.

(5) $$I=10.11\times 10^{-3}PW+6.95$$

The two neuronal activation curves were sufficiently steep and far apart that the maximum selectivity achieved was nearly unity. Had a stopping criterion been imposed on the routine, it would have stopped the search after the second iteration. Although applying Powell’s method to the case of two neurons is relatively straightforward, the true utility of Powell’s method becomes apparent in higher dimensions, such as a larger population of neurons or a greater number of stimulus parameters.

### 3.2. Experimentally Measured Strength–Duration Curves for the Neuronal Population

**Figure 7.**
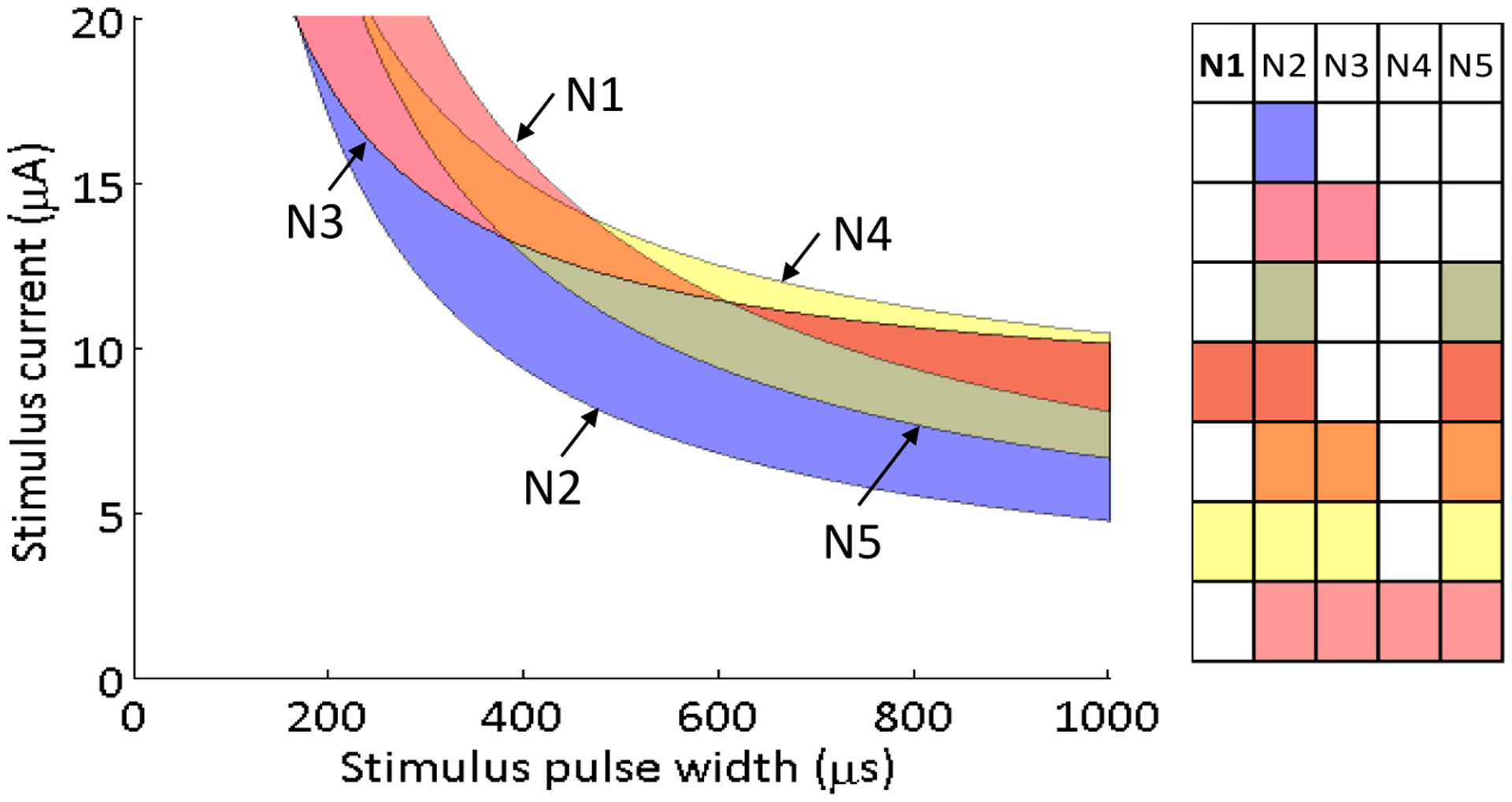
A map of selectivity regions accessible using one stimulating electrode. The strength–duration curves associated with P = 0.5 are plotted for each of the five neurons, and the regions in between the curves are color coded to define the population that is activated within that waveform region.

During the experimental implementation of the search routine for neurons N1 and N2, three additional neuronal activation curves were measured. As described previously, 50 targeted stimuli were delivered, per neuron, in each of the stimulus search directions. These stimuli were delivered in order to increase the probability measurement resolution along the transition region (0.25–0.75) of each neuron. At the conclusion of the search routine, the algorithm had collected measurements for the each of five neuronal activation curves through the strength–duration waveform space. Each sigmoid provided estimates of the 0.25, 0.50 (midpoint, or activation threshold) and 0.75 probabilities along the experimental search directions; these points were used to construct probability strength–duration curves fit to Equation (2). This means that a separate strength–duration curve was calculated for each neuron at each of the three probability levels. For other search directions through the 2-D strength–duration space, a sigmoid activation curve could be approximated by fitting the model in Equation (1) to the points where the search line intersected the 0.25, 0.50, and 0.75 probability strength–duration curves. Therefore, the sets of strength–duration curves could be used to approximate the activation probability for each neuron at any point in the strength–duration waveform space. For these five neurons, the strength–duration parameters (rheobase, *r*; chronaxie, *c*) for the 50% activation curves shown in Figure 7 are as follows: N1 (2.91 μA, 5153 μs), N2 (1.73 μA, 3046 μs), N3 (8.17 μA, 1951 μs), N4 (7.34 μA, 3079 μs), N5 (2.58 μA, 4079 μs).

All possible neuronal activation combinations were mapped in the strength–duration waveform space (Figure 7). This selectivity map shows that regardless of the goal, neuron N2 will always be activated before other neurons. The activation spaces between the four other activation curves are more nuanced because they intersect each other. The strength–duration curves cross because some neurons preferentially activate at shorter stimulus pulse widths and higher currents, while other prefer longer stimulus pulse widths and lower currents.

### 3.3. Powell’s Method Applied in Simulation to Find the Most Selective Waveform for a Population of Neurons

Simulation studies were performed in order to investigate the behavior of Powell’s method for various neuronal subpopulations. As a first example, we chose a target region within the population strength–duration space defined in Figure 7 that promotes the activation of neurons N2, N3 and N5, while suppressing the activation of neurons N1 and N4 (colored orange). The region within the stimulus waveform space that maximizes the objective function is closed. We chose this region because we predicted that it would be the most difficult region to locate using Powell’s method. The theoretical maximum of the objective function is 3, which occurs when the three target neurons are activated and the two off-target neurons are not. As the stimulus strength increases and the target neurons activate, the value of the objective function increases, but as the off-target neurons activate, the value of the function decreases. For example, if an off-target neuron activates while the three target neurons activate, then the objective function will evaluate to 2. However, if none of the target neurons activate along a particular line search, but both off-target neurons activate, then the objective function will evaluate to −2, which is the theoretical minimum. The objective function for each line search was defined according to Equation (3). For the target population N2, N3 and N5, the objective function was

(6) $$f=-P_{N1}+P_{N2}+P_{N3}-P_{N4}+P_{N5}$$

**Figure 8.**
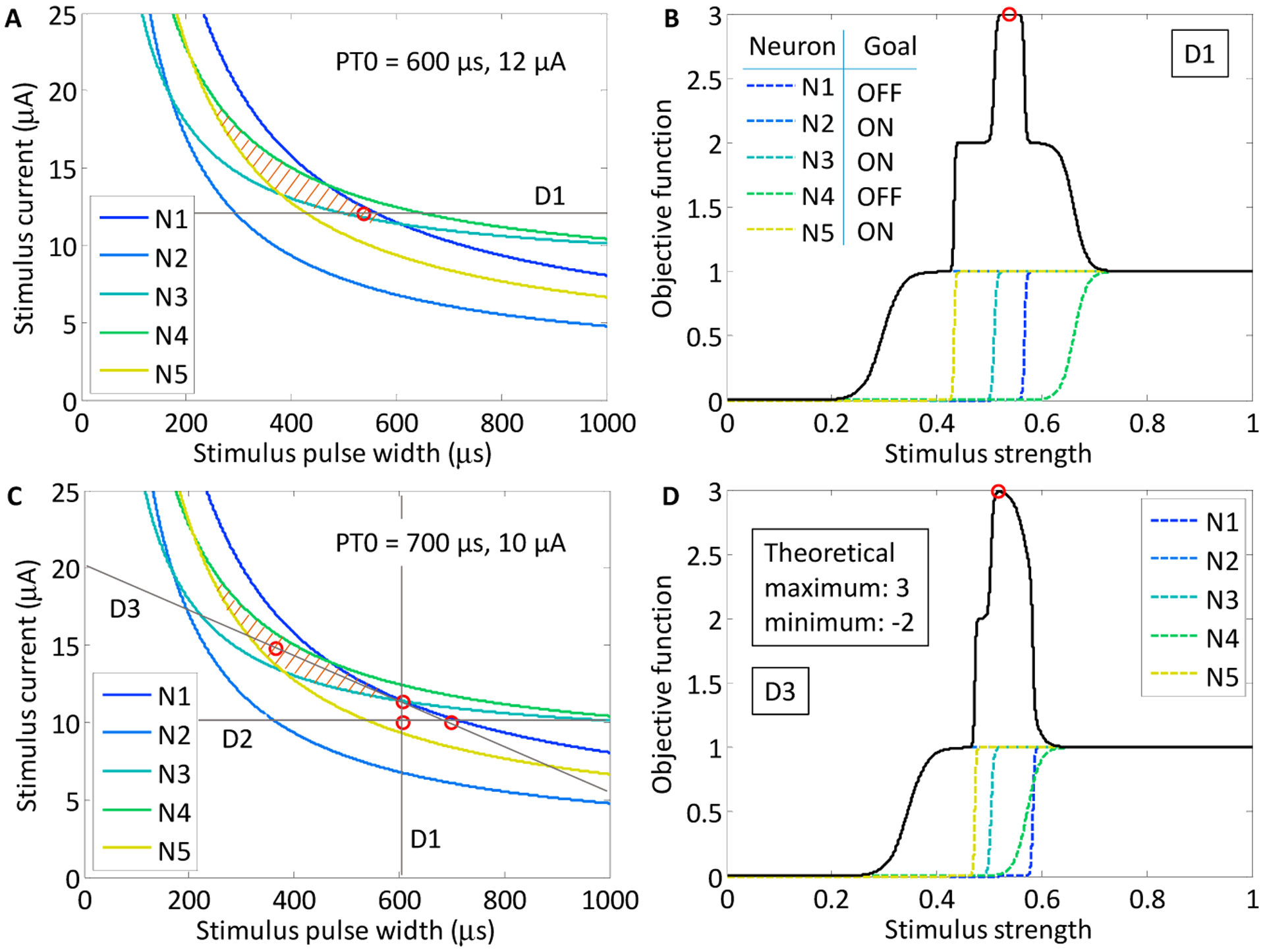
Two simulation studies were performed to find the selective region for the subpopulation of neurons including neuron N2, N3 and N5. (**A**) The selective region is highlighted with stripes. For the first study, a horizontal search through the starting point, PT0 (600 μs, 12.0 μA) yielded the theoretical maximum of the objective function (open red circle at 535 μs, 12.0 μA); (**B**) the objective function value is plotted along the first line search, D1. The individual neuronal activation sigmoids are plotted alongside the objective function (dotted lines). The first three target neurons activated before the off-target neurons activated; (**C**) The output from the second stimulation study, in which the starting point, PT0, was shifted to 700 μs and 10.0 μA; (**D**) After the 3rd line search, the theoretical maximum was found for the objective function.

As shown in Figure 8, we found that there was variation in possible outcomes of the search routine, depending on two initial conditions: the starting point, PT0, in the strength–duration space, and the orientation of the first search direction, D1. In the first study, the initial search direction was a horizontal line crossing through the point PT0 (600 μs, 12.0 μA). On the first search, the theoretical maximum of the objective function was found. This point was located in the lower corner of the target region in the SD waveform space at 535 μs and 12.0 μA.

For the second study, the starting point was shifted to a region where a line search in either direction could not yield an objective function value of 3. This point was located at 700 μs and 10.0 μA. The first horizontal line search crossed the waveform space where target neurons N2 and N5 activated first, however, neuron N3 only activated after neuron N1. The maximum of the objective function was 2. As the experimental search routine demonstrated earlier, the maximum of the objective function became the point through which the next search direction would cross. The search routine was iterated until the theoretical maximum of 3 was found at 366 μs and 14.8 μA, after search direction D3. To confirm that the result was stable in the target region, an additional three searches were run, and all results remained within in the target area.

For a second target population, we chose an objective function that promotes the activation of neurons N1, N2 and N5 (brown region in Figure 7), while penalizing the activation of neurons N3 and N4:

(7) $$f=P_{N1}+P_{N2}-P_{N3}-P_{N4}+P_{N5}$$

**Figure 9.**
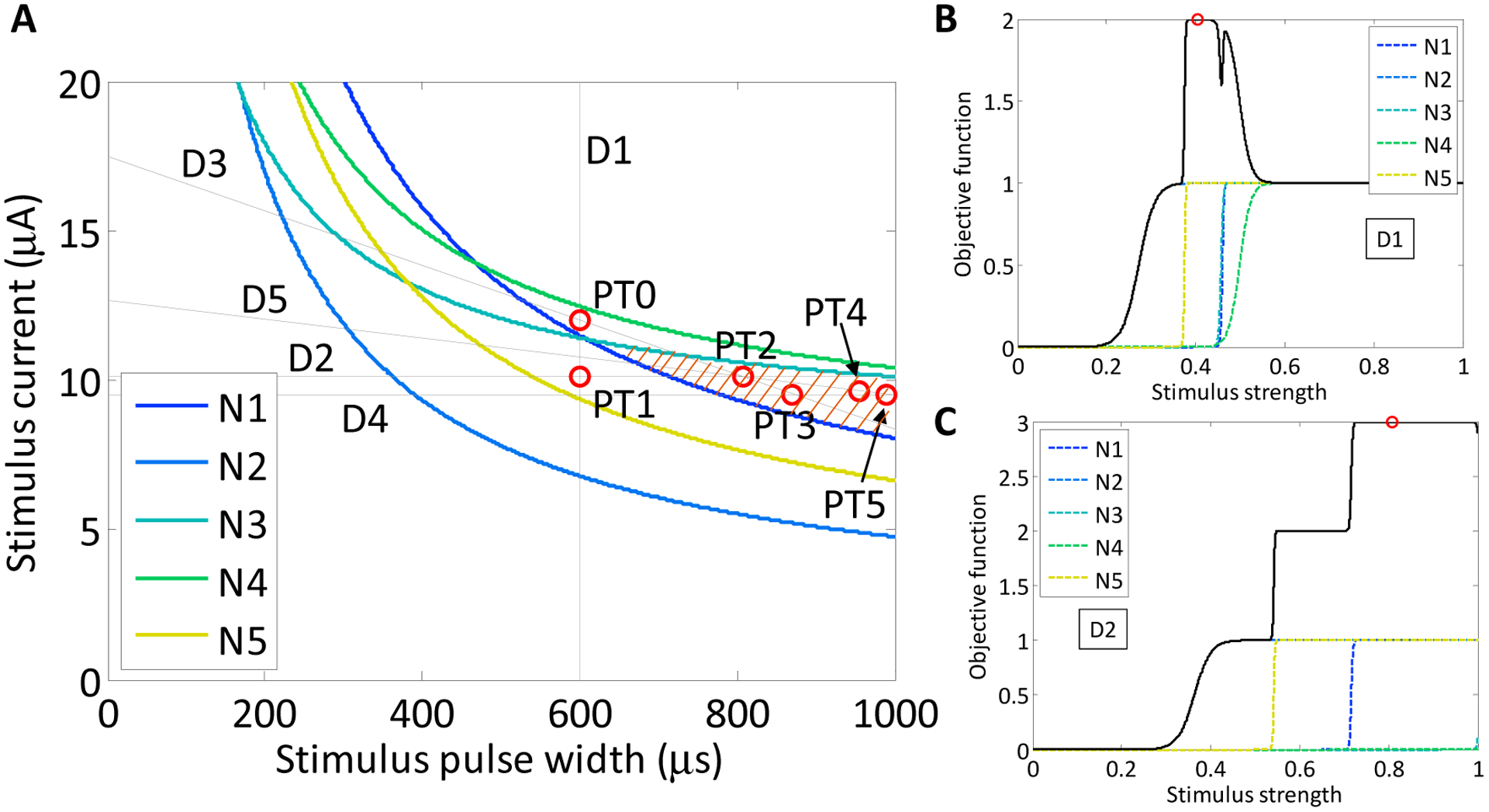
(**A**) The strength–duration curves from a fit to Equation (2) with P = 0.5, are plotted for five neurons using the experimental data collected during the CL search routine. The simulated target population of neurons was chosen, including N1, N2 and N5, and an objective function was created to promote the activation of the target neurons while penalizing the activation of the off-target neurons, Equation (7). The five searches resulting from a simulated search routine are marked with faint lines, and the objective function maxima are labeled and highlighted with open circles; (**B**,**C**) the objective function along each search direction is plotted with a solid black line. The maximum of the function is found at the open circles in each plot, which correspond to the various search directions in Powell’s method.

As in the implementation of Powell’s method for the experimental two-neuron search routine, we chose to use the starting point, PT0 (600 μs, 12.0 μA), which was located in the middle of the strength–duration waveform space, as shown in Figure 9. The first search was a stimulus current search with fixed stimulus pulse-width. The maximum of the objective function was found at PT1 (600 μs, 10.1 μA) and was approximately 2. Through this vertical search line, there was no region where all three target neurons were ON while the two off-target neurons were OFF. There existed, however, a stimulus region where two of the target neurons activated, but the third neuron would not activate until after one of the off-target neurons turned ON. As the stimulus value increased, the first two neurons activated, and the objective function evaluated to 2; then as an off-target neuron activated, the objective function decreased to 1; next the third target neuron activated, which brought the objective function close to 2 again, until the final off-target neuron activated and pulled the objective function back down to 1. At the highest allowed stimulus value, the function would always evaluate to one.

The next search was simulated in the variable-pulse-width, constant-current direction. Again a sigmoid model was constructed for each neuron and the objective function was evaluated. The maximum was 3, the maximum that was theoretically possible, and was found at PT2 (807 μs, 10.1 μA). Although the maximum was found after the second search, the routine was continued to evaluate its stability. The third search was along the line that connected PT0 and PT2. In this search direction, the models constructed for N1, N2 and N5 were single positively-sloped sigmoids because the slice only crossed the left side of the neurons’ set of SD curves, similarly to a horizontal search. The model constructed for N3 was a set of two sigmoids (described in Figure 4D) and for N4 was zero because the slice did not cross the set of SD curves for that neuron. The maximum of the objective function, combining all five neuronal activation models according to (7) was found at PT3 (870 μs, 9.5 μA). The fourth search was horizontal, parallel to the second search, and the maximum of the function was found at PT4 (990 μs, 9.5 μA). Finally, the fifth search was again in a negative slope direction and the maximum selectivity was found at PT5 (954 μs, 9.6 μA).

As the search progressed, the waveform at the maximum selectivity shifted toward long-pulse-width stimuli. As is observable within the set of SD curves, at longer pulse widths, the activation of neurons N2 and N4 converge toward a higher level, while neurons N1, N2 and N5 all tend lower. This produces a selectivity region exhibiting larger stimulus pulse widths.

## 4. Discussion

### 4.1. The Choice of Powell’s Method

Deterministic search methods, like Powell’s Method, have a great strength in optimization because they converge quickly with a good initial starting point; the downside of deterministic methods is that the search can be trapped in local minima if a poor initial starting point is used. A gradient-based search method, such as gradient descent, is an undesirable method for finding the selective region between populations of neurons because there exists a plateau of maximum selectivity between neuronal strength–duration curves. Additionally, a gradient-based search routine is susceptible to instability when applied to noisy data. Other non-gradient search methods could conceivably be implemented for optimizing neuronal stimulation parameters including, Nelder-Meade, simulated annealing, or a genetic algorithm. The significance of this experiment was to demonstrate the feasibility of an optimization method applied directly to the experiment. In the experimental implementation, the neuronal activation curves were relatively far apart in the strength–duration waveform space, resulting in a large region between the two P = 0.5 curves where selectivity of neuron N2 over N1 is high. For that reason, the algorithm was able to quickly converge and find a stimulus solution where the absolute value of the difference in activation sigmoids was approximately 1, the theoretical maximum of the two-neuron objective function, after only two search iterations.

Methods for global optimization of deterministic systems are fairly advanced [40], but they require a lot of function evaluations, and usually this number is many more than could be feasibly delivered in a biological experimental setting. For stochastic systems like the neuronal system presented here, the problem is much more difficult [41]. Local optimization methods are often used in the stochastic experimental context, although global optimization could be used in principle. Optimization routines such as these that require a very large number of evaluations are often implemented in non-biological or simulation environments, which is impractical for the experimental limitations presented here. Biological models often require many parameter estimations, and the problem of using experimental data in optimization often comes up in parameter estimation for biological systems [42]. While another approach may benefit from the implementation of a different optimization method and search technique, the use of Powell’s Method should be extendible to more parameters to include a more complex stimulus space, e.g., additional parameters such as stimulus frequency, and a larger population of neurons.

### 4.2. Weighting the Objective Function

As the objective function complexity increases, there is an increasing likelihood that the theoretical maximum of the objective function is unachievable. For these cases, the goal is to find the stimulus that will be most selective for one subpopulation over another. For example, there is no perfect waveform region for an objective function that targets neurons N1, N2 and N3 while not activating neurons N4 and N5 (Figure 7). However, the objective function is still higher in some regions of the space, compared to others. The objective function would evaluate to 2 in the region where neurons N2 and N3 are fully activated (P1 + P2 + P3 − P4 − P5 = 0 + 1 + 1 − 0 − 0) because there is a penalty for not activating neuron N1. In the waveform space where neurons N1, N2, N3 and N5 activate, the objective function would again evaluate to 2 (P1 + P2 + P3 − P4 − P5 = 1 + 1 + 1 − 0 − 1). In this waveform region, all three target neurons were activated, but there was a penalty because the off-target neuron N5 was also activated. The preference for one waveform region over another will require additional factors to be included into the objective function. Various neuronal probability combinations will result in the same function evaluation without the inclusion of weighting to emphasize an experimental goal of activating a particular set of neurons. For example, in a three neuron set, activation of neuron 1 and 2, or neuron 1 and 3, or neuron 2 and 3 would all evaluate to the same value if all three neurons were equally weighted. However, the objective function could emphasize the activation of on-target neurons by applying additional weight to P1, P2 and P3. This weighting would bias toward the second waveform region, in which all three target neurons are activated because the increase in the objective function from activating the third neuron would outweigh the penalty for activating the off-target neuron. Conversely, the objective function could minimize off-target activation by applying an increased penalty for activating off-target neurons. This increased penalty would naturally select the first waveform region, in which only neurons N2 and N3 are activated, because the penalty for activating neuron N5 would outweigh the benefit of activating neuron N1.

For an in vivo study the objective function may be governed by a larger population, for example, a study’s goal could be to maximize the beneficial desired behavior while minimizing side effects. The evaluation of a function in an experiment like that may weigh the activation intensity of an entire neuronal subpopulation. A similar search routine could be implemented in which there is defined a maximum stimulus that is allowable due to both safety precautions and due to the complementary activation of an unwanted larger population than is desired. That maximum stimulus could then be used to bracket the stimulus waveform space and a search could be performed in which wanted and unwanted populations are weighted differently.

### 4.3. Choosing the Selectivity Point after Each Iteration

We chose an objective function that maximized the difference of sigmoids. However, this function is susceptible to sudden shifts in the output when there is a plateau between the neuronal activation curves, because the value of the objective function changes very slightly across the plateau. For example, given a pair of neurons in which one has a very steep activation curve and the other has a shallower curve, the maximum of the difference in sigmoids will occur very close to the steep neuron’s transition region. This result occurs because the steep activation curve will evaluate to nearly one very near to the transition region, while the shallower sigmoid will more slowly transition from zero to one; the maximum of the objective function will appear as far from the shallower sigmoid as possible. The resulting maximum of the objective function occurring so close to the steep neuronal activation curve may not be the ideal place to stimulate for selectivity. It may be preferable instead to stimulate closer to the midpoint of span of stimuli that produce difference in sigmoids, as quantified by *f*, above a predetermined fixed value. In selecting a stimulus at the midpoint of the span, the chosen stimulus will maximize the distance between the selected neurons. As a selectivity point, the midpoint will have greater robustness because any variation in the internal state of either neuron is less likely to cause the neuronal activation probability to deviate significantly. Accounting for this factor is of greater import for longer term studies or those in which synaptic blockers are not used, where drifting of the culture is more likely to occur.

### 4.4. Limitations in the Approach

The advantage of simulation studies is the sheer number of function evaluations that are possible where those possibilities are extremely limited in a biological experimental system. Further studies will be necessary to elaborate on, and advance, the techniques presented here. In this work, we are proposing and demonstrating a new concept and approach, however the specific simulation studies performed in this work were not directly validated with experimental testing due to the limitations inherent in an experimental approach.

We utilized an in vitro model neuronal system to specifically study only the direct activation of neurons. While other systems, including in vivo neuronal structures, may not have the simplicity of a synaptically silenced system like that which is presented here, we are interested in studying the way in which stimulus waveforms directly evoke activity in a given neuron. By eliminating down-stream synaptic communication in the model culture, we have essentially made a black box of the network. This then allows us to assume, for example, that in a scenario where neuronal communication is in tact downstream the expressed purpose of delivering stimuli through an array of micro-electrodes in only to activate an initial target neuron in a culture. In this study, the neurons were uncoupled from the surrounding network using synaptic blockers. While the CL system can estimate a neuronal activation threshold, the algorithm will require modification for application to tracking potentially non-stationary activation curves in a coupled network. In the studies presented herein, neuronal activation curves proved to be stable over the experiment, however, a particular neuron’s activation curve may not be stationary in the presence of synaptic network input.

## 5. Conclusions and Future Directions

In this work we demonstrated that a closed-loop search routine implemented according to Powell’s method could be used to find the waveform region that is most selective for a subpopulation of accessible neurons. We used a model-based search method for optimizing stimulus parameters in the strength–duration space to target an arbitrary set of neurons. The success of this technique is attributable to exploiting the natural variation in strength–duration curves between neurons. In our experimental system, we use wide-field optical imaging as a measurement tool; it is likely that in other applications non-optical methods will be used to record evoked activity. The findings in this work are independent of measurement method, and so also apply to non-optical recording methods. Ultimately, any stimulation routine needs to implement a technique to probe and characterize the population response in order to design targeted stimuli that will enable more sophisticated control of the evoked response. In the experimental application and in biological systems in general, there is variability in population size, absolute neuron position, and relative position of the cells to the micro-electrode arrays. It must therefore be assumed that each experimental application will have a unique response. It is the uniqueness of each application that requires that the accessible neuronal population be learned, and this accessible population be probed for response in the stimulus parameter space.

While CL systems, such as the one presented here, enable learning about the nervous system, they are also essential for clinical applications. For example, in delivering sensory stimuli from prosthesis back to the brain, message encoding algorithms must be developed that measure evoked activity online. Online feedback of the evoked activity will enable the controller to find the most separable stimuli. Closed-loop techniques are indispensable for guiding a stimulus to be most efficacious in a neuronal environment. In order to control the activity of a particular population, it must be characterized online to measure how it is changing and evolving with each stimulus presentation.

The use of a model-based closed-loop search routine shows greatest benefit in larger dimensional spaces. Multiple stimulating electrodes can be used to further increase selectivity; each additional electrode doubles the dimension of the input parameter space. Future studies will examine the increase in selectivity achievable using multiple electrodes and more complex stimulus waveforms, which will result in even higher dimensional spaces. Future medical devices will use many electrodes in order to encode more complex messages, which will require optimization routines similar to the work presented here, which are effective in higher dimensional spaces. Although the Powell search routine was implemented in this work, other search methods could be implemented including Nelder-Meade, simulated annealing, or a genetic algorithm. These alternative search algorithms may offer advantages over Powell’s method depending on the specific stimulation configuration and recording environment.
